# Fc-engineered monoclonal antibodies to reduce off-target liver uptake

**DOI:** 10.1186/s13550-023-01030-0

**Published:** 2023-09-11

**Authors:** Tristan Mangeat, Matthieu Gracia, Alexandre Pichard, Sophie Poty, Pierre Martineau, Bruno Robert, Emmanuel Deshayes

**Affiliations:** 1 Institut de Recherche en Cancérologie de Montpellier (IRCM), Inserm U1194, Université de Montpellier, ICM, 34298 Montpellier, France; 2 Institut Régional du Cancer de Montpellier (ICM), Service de Médecine Nucléaire, 34298 Montpellier, France; 3 Institut de Recherche en Cancérologie de Montpellier (IRCM), 124 Avenue des Apothicaires, 34090 Montpellier, France

**Keywords:** PET-CT, Fc gamma receptor, Off-target, Fc, LALAPG mutation

## Abstract

**Background:**

Radiolabeled-antibodies usually display non-specific liver accumulation that may impair image analysis and antibody biodistribution. Here, we investigated whether Fc silencing influenced antibody biodistribution. We compared recombinant ^89^Zr-labeled antibodies (human IgG1 against different targets) with wild-type Fc and with mutated Fc (LALAPG triple mutation to prevent binding to Fc gamma receptors; FcγR). After antibody injection in mice harboring xenografts of different tumor cell lines or of immortalized human myoblasts, we analyzed antibody biodistribution by PET-CT and conventional biodistribution analysis.

**Results:**

Accumulation in liver was strongly reduced and tumor-specific targeting was increased for the antibodies with mutated Fc compared with wild-type Fc.

**Conclusion:**

Antibodies with reduced binding to FcγR display lower liver accumulation and better tumor-to-liver ratios. These findings need to be taken into account to improve antibody-based theragnostic approaches.

**Supplementary Information:**

The online version contains supplementary material available at 10.1186/s13550-023-01030-0.

## Introduction

Off-target binding of drugs is a common problem in diagnostic and therapeutic settings. This is also the case for antibodies, although unlike other drugs, they bind to a specific target mainly due to the exquisite specificity of their antigen-binding fragment. In oncology, this sought-after high specificity can induce off-tumour binding because, to date, most antibody targets are over-expressed in tumour tissue (on-target, on-tumour), but they can also be expressed at basal levels in healthy tissue (on-target, off-tumour). In addition, the crystallizable (Fc) region of the antibody fragment can also induce off-target binding by interacting with Fc gamma receptors (FcγR), which are expressed in many tissues, in particular by liver Kupffer cells in addition to being expressed on the surface of cells of the immune system. To counteract this, certain antibodies are now selected with a lower specific affinity to reduce their off-target binding [1] or by re-engineering [2]. Concerning the Fc domain, mutations N297-A or -D, L234A/L235A (LALA), G236R/L328R (RR), S298G/T299A (GA) and S228P/L235E (IgG4-PE) significantly reduce binding to Fc gamma receptors (FcγRI and FcγRIIA) [3–5], and the triple mutation L234A/L235A/P329G (LALAPG) significantly reduces binding to all FcgRs [5]. Several studies have shown that the introduction of the N297A or S228/L235E mutations has no impact on the tumour localization of several antibodies but does allow a longer half-life for antibodies carrying these mutations via reduced catabolism by the liver [6–8]. In this study, we compared the biodistribution of ^89^Zr-labelled antibodies carrying the LALAPG triple mutation (Fc-LALAPG) with the same antibodies carrying a wild-type Fc, as these triple mutations are even more potent than N297D mutation in abolishing FcγR binding and some clinical trials using mAbs incorporating these triple mutations are in progress [5].

## Materials and methods

### Recombinant antibody production

D4a mAb (patent number WO2016091891A1) is specific to human AXL tyrosine kinase receptor since it does not bind to mouse Axl and other human receptors of the same family, TYRO3 or MER tyrosine kinase receptors. 13R4a mAb is specific of *E. coli* beta-galactosidase specific and this antibody is used as an isotype irrelevant control [9]. Interestingly, the synthetic library is build using 13R4a clone as template, meaning that there are only few differences between D4a and 13R4a exclusively located in the 6 CDR loops. These two mAbs have been isolated in the laboratory from a human synthetic library of scFv using phage display [10]. ScFv were reformatted as full human IgG1 with wild type (WT) Fc by cloning variable heavy and light chain in an expression plasmid. The L234A/L235A/P329G mutations were introduced in coding plasmid by targeted PCR-mutagenesis and validated by sequencing. 13R4a and D4a antibodies with wild type Fc and with the L234A/L235A/P329G mutation (LALAPG Fc) were produced in HEK293T cells by transient transfection with polyethylenimine. Antibodies were purified from supernatant using Protein-A agarose beads and dialyzed against PBS. Purity was verified on SDS-PAGE. Antibody binding was validated in vitro by enzyme linked immunosorbent assay and by fluorescent-activated cell sorting in different cell lines.

### Radioimmunoconjugation

Antibodies (human IgG format) were functionalized with pSCN-Bn-deferoxamine in a non-site-specific manner before radiolabeling with ^89^Zr. Briefly, the antibody buffer was exchanged to chelexed PBS using Amicon® Ultra Centrifugal filters (30 kDa cut-off). pH was adjusted to 8.5–9.0 using 0.2 M chelexed Na_2_CO_3_, and a 15-fold excess of pSCN-Bn-deferoxamine was added to the solution (1.6–2.1 mg/mL, 500 µL). After incubation at 37 °C with gentle shaking for 60 min, excess pSCN-Bn-deferoxamine was removed using Amicon® Ultra Centrifugal filters as before. [^89^Zr] Zr-oxalate (Perkin Elmer) (30 MBq) was neutralized to pH 6.9–7.2 with 1 M chelexed Na_2_CO_3_ before addition of the deferoxamine-immunoconjugates and incubation at room temperature with gentle shaking for 1 h. Purity and radiolabeling efficiencies were determined using instant thin-layer chromatography with 0.1 M sodium citrate (pH 5.0) as mobile phase. Radiolabeling yield and radiochemical purity were routinely > 99%. No purification step was performed. Radioimmunoconjugates had a specific activity of 200 MBq/mg and were formulated in 0.9% NaCl for in vivo use. Analysis of the immunoconjugate was performed by Maldi-tof (Rapiflex, Bruker) to determine the number of DFO conjugated to mAbs. The number of DFO molecules is determined by dividing the difference in the m/s ratio of the peak of the whole antibody conjugated with DFO and unconjugated with the molecular weight of DFO (752 Da) (Additional file 1: Figure S1). Binding affinity of D4a mAbs conjugated with DFO was performed by flow cytometry using AXL expressing cell line. A binding affinity experiment was presented in Additional file 1: Figure S2.

### Cell lines and mice

The AXL-expressing [11, 12] human MDA-MB-231 (triple negative breast cancer) and CFPAC-1 (pancreatic cancer) cell lines were from American Type Culture Collection. Immortalized human myoblasts (provided by Vincent Mouly, UMR-S 974) express low AXL level and were used to mimic healthy human tissues. Cancer cells were cultured in Dulbecco’s Modified Eagle’s Medium (DMEM) with 10% Fetal Bovine Serum (FBS, Eurobio), and myoblasts in skeletal muscle cell growth medium (C-23060, Promocell) with 20% FBS at 37 °C and 5% CO_2_. All animal experiments were performed in compliance with the European directive (2010/63/EU) and the INSERM standards for experimental animal studies (agreement E34-172-27). They were approved by the Institut de Recherche en Cancérologie de Montpellier (IRCM/INSERM U1194) and the Languedoc Roussillon region (CEEA LR France No. 36) ethics committees. Cells (5.10^6^) in Matrigel (Corning) were injected subcutaneously in 6-week-old female athymic nude mice (Crl: Nu (NCr)-Foxn1nu, Charles River).

### Imaging

Four weeks after cell xenografts, [^89^Zr] Zr-deferoxamine-labeled antibodies (50 µg, 200 MBq/mg) were injected in the tail vein and in vivo images were acquired with a Mediso NanoScan PET82S/CT80 system at 48, 72, and 96 h post-injection. Anesthesia was induced with 4% isoflurane in air followed by maintenance with 2% isoflurane in air. Images were acquired with Nuclide and processed with Interview™ FusionTM. Volumes of interests were manually drawn on fused PET-CT images using 3D Slicer. Results are expressed as percentage of the injected activity per cm^3^ (%IA/cm^3^).

### Conventional biodistribution analysis

Biodistribution was assessed at 96 h post-injection after the last imaging time-point. After euthanasia, tumor, myoblast xenografts, and liver were excised, weighted, and activity counted with a gamma-counter (Hidex AMG) together with standards of the injected radiolabeled antibodies. Results were expressed as percentage of the injected activity per gram of tissue (%IA/g).

## Results

### Imaging

**Fig. 1:**
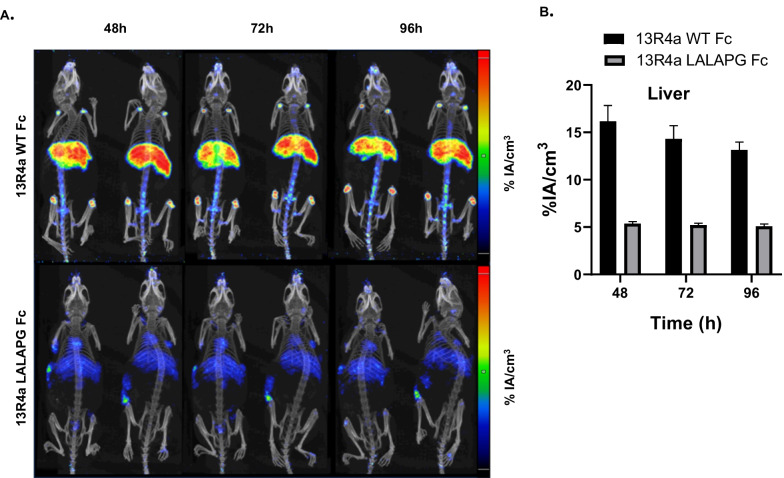
Liver accumulation of a bacterial anti-beta galactosidase IgG is reduced by the LALAPG triple mutation. **A** Maximal Intensity Projection (MIP) PET images of mice subcutaneously xenografted with myoblasts (left flank) at 48, 72 and 96 h after the injection of 89Zr-radioimmunolabeled 13R4a with LALAPG Fc or with WT Fc (*n* = 2 mice per group). The low and high values correspond to 1 and 5% IA/cm3 respectively and are indicated by a low and high line on the scale. **B** Quantitative PET analysis (PET based) of antibody accumulation in liver at different times post-injection (48 h, 72 h and 96 h) expressed in %IA/cm3

**Fig. 2:**
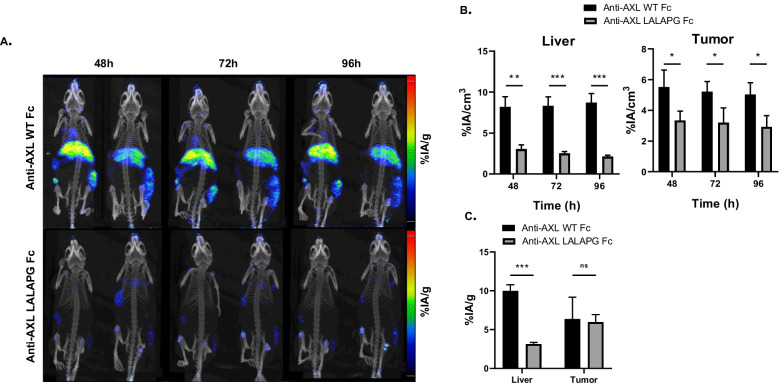
Anti-AXL D4a IgG with LALAPG Fc displays lower liver retention than the IgG with WT Fc. **A** Maximal Intensity Projection PET images of mice subcutaneously xenografted with myoblasts (left flank) and tumor cells (right flank; CFPAC-1 cells for the IgG with WT Fc and MDA-MB-231 cells for the IgG with LALAPG Fc) at 48, 72 and 96 h after injection of ^89^Zr-radioimmunolabeled anti-AXL D4a IgG with LALAPG Fc or WT Fc (*n* = 3 mice per group). The low and high values correspond to 1 and 5% AI/g respectively and are indicated by a low and high line on the scale. **B** Quantitative PET analysis of anti-AXL IgG in liver at 48 h, 72 h and 96 h post-injection, expressed in %IA/cm^3^. **C** Conventional biodistribution analysis of the anti-AXL IgG in liver and tumors (CFPAC-1 cells for the IgG with WT Fc and MDA-MB-231 cells for the IgG with LALAPG Fc) at 96 h post-injection. The percentage of activity in the organ (%IA/*g*) corresponds to the injected activity related to the organ weight. For panels **B** and **C**: *p ≤ 0.05, **p ≤ 0.01, ***p ≤ 0.001; ns, not significant (unpaired two-tailed *t* test)

First, we studied the biodistribution of the 13R4a IgG against bacterial ß-galactosidase that is not expressed in mice to avoid the effect of the targeted antigen. To respect the 3R rules, we used mice previously xenografted with immortalized myoblasts (left flank). At 48, 72 and 96 h after injection of ^89^Zr-radiolabeled 13R4a antibodies with wild-type Fc or LALAPG Fc, PET images showed a strong accumulation of 13R4a with wild-type Fc in liver (Fig. 1A, up) and a weak accumulation of 13R4a with LALAPG Fc (Fig. 1A, down). We observed accumulation in joints (shoulder and knee) particularly of the wild-type antibody, possibly explained by the natural tropism of zirconium 89 for bones and joints [13]. Quantification of liver uptake confirmed a decrease in liver accumulation by threefold at 48 h (5.4 vs 16.2%IA/cm^3^) and by twofold at 96 h (5.1 vs 13.1%IA/cm^3^) of 13R4a-LALAPG Fc compared with 13R4a-wild-type Fc (Fig. 1B). As 13R4a does not recognize any target in mice, we then used D4a, an antibody against human (but not mouse) AXL, and three human cell lines with different AXL expression: immortalized myoblasts (low AXL expression) and MDA-MB-231 and CFPAC cancer cells (high AXL expression). We injected ^89^Zr-radiolabeled D4a with wild-type Fc in mice xenografted with myoblasts (left flank) and with CFPAC-1 cells (right flank) and ^89^Zr-radiolabeled D4a with LALAPG Fc in mice xenografted with myoblasts (left flank) and MDA-MB-231 cells (right flank). We could not quantify antibody accumulation in myoblast xenografts due to spatial resolution and xenograft size limitations. Like with 13R4a, D4a with wild-type Fc strongly accumulated in liver at 48, 72 and 96 h post injection (Fig. 2A, up), but not D4a with LALAPG Fc (Fig. 2A, down). Quantification at 96 h confirmed the significantly lower liver retention of D4a with LALAPG Fc than with wild-type Fc (2.1 ± 0.2 vs 8.7 ± 1.1%IA/cm^3^; *p* < 0.001) (Fig. 2B). Our findings demonstrate that the LALAPG mutation in the Fc region reduced drastically the liver retention of two antibodies (D4a and 13R4a).

### Biodistribution analysis

The biodistribution study showed a reduced (by fourfold) liver accumulation of ^89^Zr-radiolabeled D4a with LALAPG Fc compared with wild-type Fc at 96 h (3.1 ± 0.2 vs 10.0 ± 0.8%IA/g; *p* < 0.001) (Fig. 2C). Accumulation in CFPAC-1 and MDA-MB-231 tumors was modest for both D4a with wild type and with LALAPG Fc (6.3 ± 2.8 and 6.0 ± 1.0%IA/g). However, the tumor-to-liver ratio for D4a with LALAPG Fc was 1.9 (vs 0.6) thanks to the very low liver accumulation.

## Discussion

We demonstrated that the LALAPG triple mutation in the Fc region strongly reduces accumulation in liver of ^89^Zr-radiolabeled antibodies. Liver accumulation of metal-radiolabeled antibodies is frequently observed in diagnostic and therapeutic settings [14, 15]. A previous biodistribution study showed that an afucosylated IgG promotes higher liver accumulation mediated by FcγR binding [7]. Reducing this binding, without affecting the antibody half-life, could decrease liver accumulation. Therefore, we used antibodies harboring a triple Fc mutation (LALAPG) that negatively affects binding to all murine FcγR [16]. Dekkers et al. demonstrated a similar binding profile of human IgG1 and mouse IgG2a to mouse FcγR [17]. The very low liver accumulation of human IgG1 with LALAPG Fc in our study can be explained by reduced interactions with mouse FcγR on hepatocytes and resident macrophages [6, 18]. This lower liver accumulation should allow antibody redistribution and favor their accumulation in tissues/tumors that express the targeted antigen. Another study showed that the N297A mutation in the Fc region of avelumab hampers FcγR binding and improves its plasma half-life in monkeys [19]. Moreover, deglycosylation (to impair FcγRI binding) of an immunoconjugate reduced the off-target uptake and increased the tumor uptake [8].

The LALAPG Fc mutation could be used to optimize therapy or diagnosis by reducing liver off-target uptake. For imaging purposes, decreasing the unspecific liver uptake is especially valuable to reinforce the contrast of liver tumors/metastases. With therapeutic (alpha, beta particles) emitters, reducing liver off-target accumulation would diminish unspecific liver irradiation. We need now to confirm the hypothesis that reducing off-target liver accumulation improves the antibody biodistribution, resulting in higher tumor uptake.

## Conclusion

We reduced non-specific liver accumulation by using antibodies harboring the LALAPG mutation in the Fc region. This could be a strategy to optimize the targeting specificity of radioimmunoconjugates used for diagnostic and therapeutic purposes.

### Supplementary Information

**Additional file 1. Figure S1:** Example of mass-analysis of mAbs conjugated with DFO. Analysis of the immunoconjugate was performed by Maldi-tof (Rapiflex, Bruker) to determine the number of DFO conjugated to mAbs. Peaks of the entire antibody are indicated by an arrow. **Figure S2:** Binding of D4a WT Fc and D4a LALAPG Fc after DFO conjugation on cancer cells by flow cytometry. Anti-AXL antibody D4a with WT Fc and with LALAPG mutations were used at 5 μg/ml to stain AXL positive cell line for 1h at 4°c in PBS-BSA buffer. After 3 wash, a secondary fluorescent labeled anti-hFc mAbs labeled was used to reveal binding of D4a mAbs at the cell surface of the cell.

## Data Availability

The datasets used and/or analyzed during the current study are available from the corresponding author on reasonable request.

## Abbreviations

^89^Zr: Zirconium 89
DMEM: Dulbecco’s Modified Eagle’s Medium
FBS: Fetal Bovine Serum
Fc: Fragment crystallizable
FcγR: Fc gamma receptors
IgG: Immunoglobulin G
PBS: Phosphate buffered saline
PEI: Polyethylenimine
PET-CT: Positron emission tomography-computed tomography
WT: Wild type
