# Supplementary material for: Fc-engineered monoclonal antibodies to reduce off-target liver uptake
Source: EJNMMI Res. 2023 Sep 11;13:81. doi: 10.1186/s13550-023-01030-0 (PMC10495296; doi:10.1186/s13550-023-01030-0)
Supplement: Supplementary file 1 — Additional file 1. Figure S1: Example of mass-analysis of mAbs conjugated with DFO. Analysis of the immunoconjugate was performed by Maldi-tof (Rapiflex, Bruker) to determine the number of DFO conjugated to mAbs. Peaks of the entire antibody are indicated by an arrow. Figure S2: Binding of D4a WT Fc and D4a LALAPG Fc after DFO conjugation on cancer cells by flow cytometry. Anti-AXL antibody D4a with WT Fc and with LALAPG mutations were used at 5 μg/ml to stain AXL positive cell line for 1h at 4°c in PBS-BSA buffer. After 3 wash, a secondary fluorescent labeled anti-hFc mAbs labeled was used to reveal binding of D4a mAbs at the cell surface of the cell. [file 13550_2023_1030_MOESM1_ESM.pptx]

## Slide 1
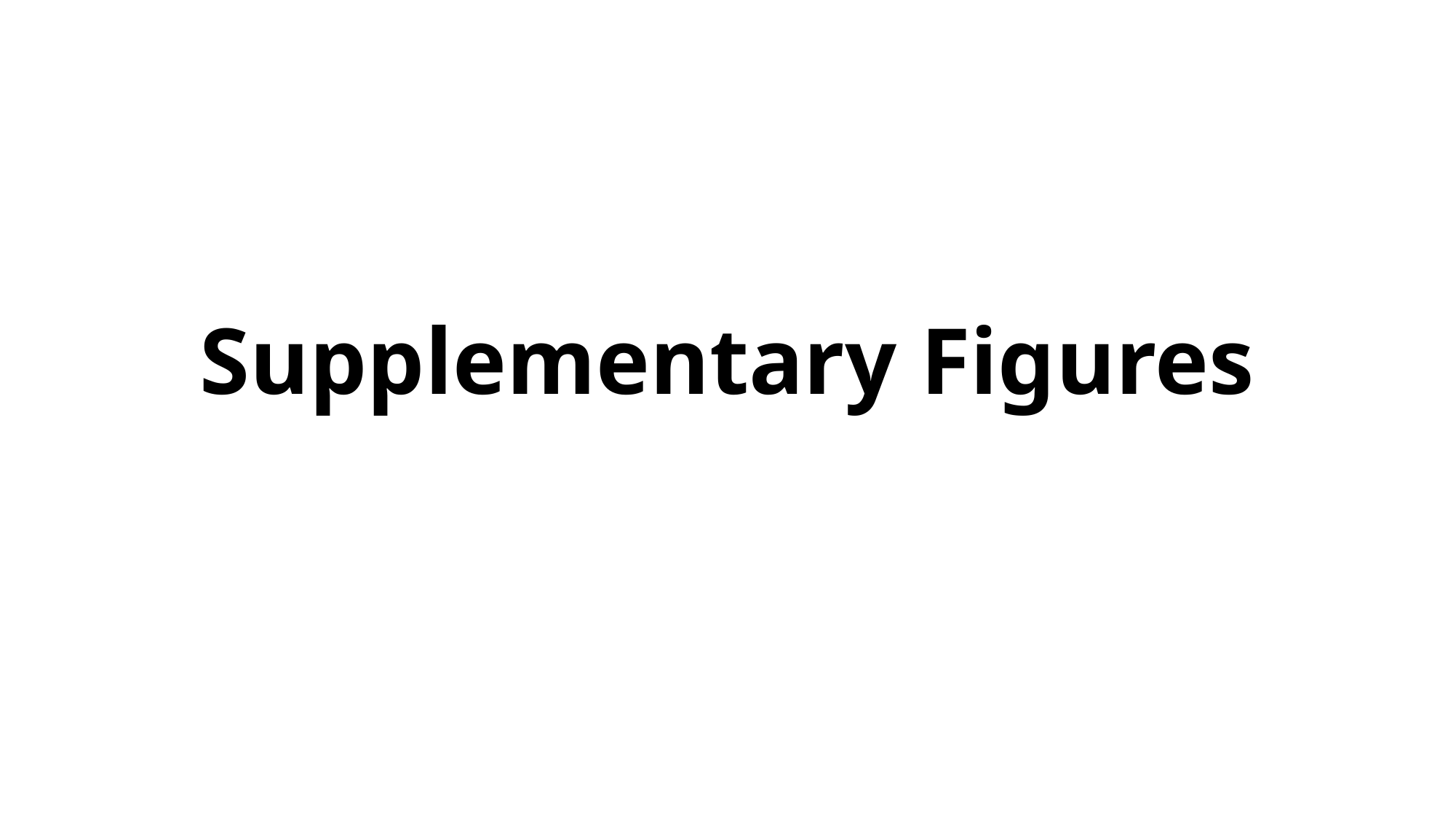

# Supplementary Figures

## Slide 2
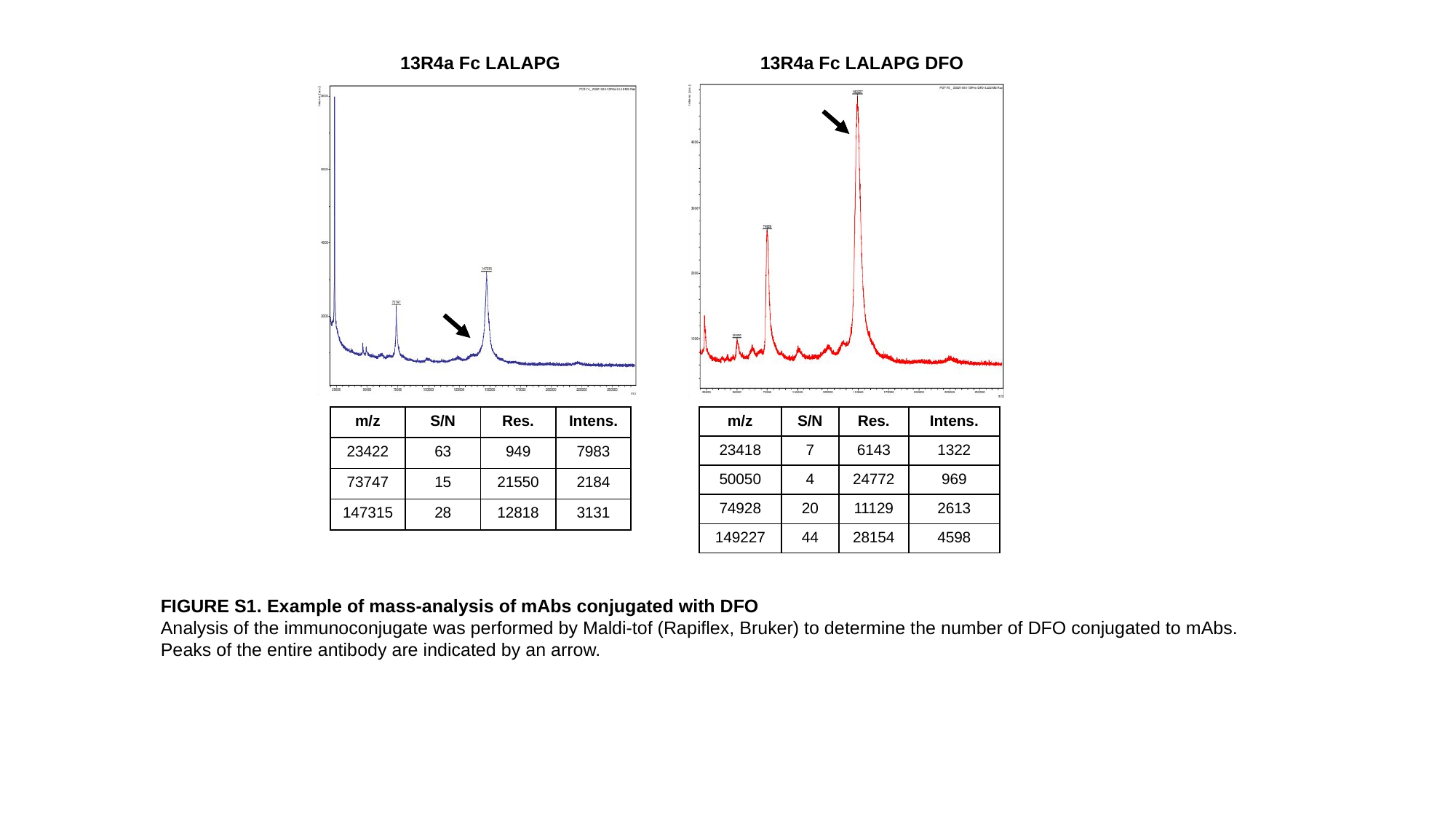

13R4a Fc LALAPG
13R4a Fc LALAPG DFO
| m/z | S/N | Res. | Intens. |
| --- | --- | --- | --- |
| 23422 | 63 | 949 | 7983 |
| 73747 | 15 | 21550 | 2184 |
| 147315 | 28 | 12818 | 3131 |
| m/z | S/N | Res. | Intens. |
| --- | --- | --- | --- |
| 23418 | 7 | 6143 | 1322 |
| 50050 | 4 | 24772 | 969 |
| 74928 | 20 | 11129 | 2613 |
| 149227 | 44 | 28154 | 4598 |
FIGURE S1. Example of mass-analysis of mAbs conjugated with DFO
Analysis of the immunoconjugate was performed by Maldi-tof (Rapiflex, Bruker) to determine the number of DFO conjugated to mAbs.
Peaks of the entire antibody are indicated by an arrow.

## Slide 3
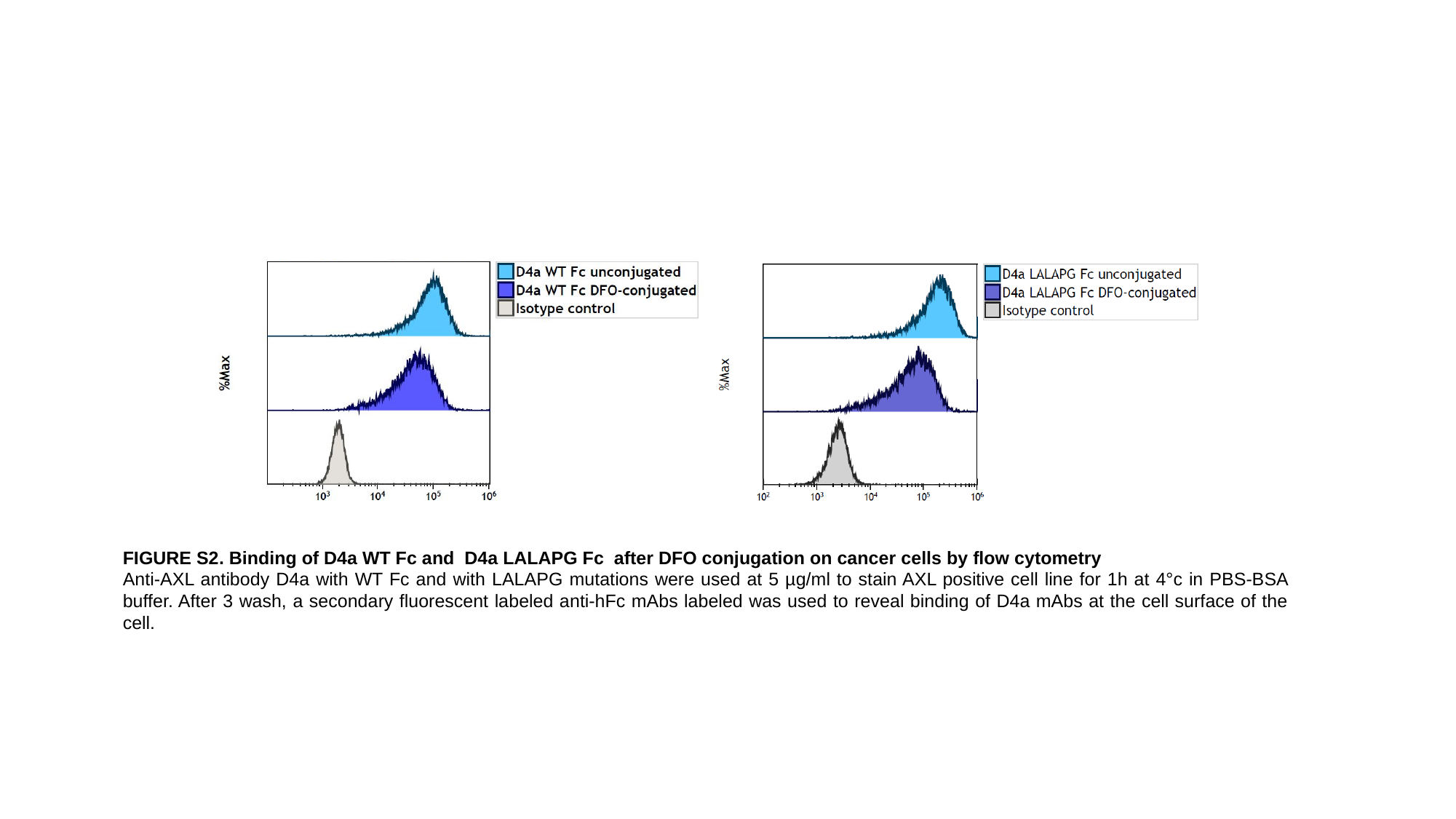

FIGURE S2. Binding of D4a WT Fc and D4a LALAPG Fc after DFO conjugation on cancer cells by flow cytometry
Anti-AXL antibody D4a with WT Fc and with LALAPG mutations were used at 5 µg/ml to stain AXL positive cell line for 1h at 4°c in PBS-BSA buffer. After 3 wash, a secondary fluorescent labeled anti-hFc mAbs labeled was used to reveal binding of D4a mAbs at the cell surface of the cell.
